# Absence of Biomarker-Driven Treatment Options in Small Cell Lung Cancer, and Selected Preclinical Candidates for Next Generation Combination Therapies

**DOI:** 10.3389/fphar.2021.747180

**Published:** 2021-08-31

**Authors:** Nicholas R. Liguori, Young Lee, William Borges, Lanlan Zhou, Christopher Azzoli, Wafik S. El-Deiry

**Affiliations:** ^1^ Lewis Katz School of Medicine, Temple University, Philadelphia, PA, United States; ^2^ Laboratory of Translational Oncology and Experimental Cancer Therapeutics, Warren Alpert Medical School, Brown University, Providence, RI, United States; ^3^ Department of Pathology and Laboratory Medicine, Warren Alpert Medical School, Brown University, Providence, RI, United States; ^4^ Joint Program in Cancer Biology, Lifespan Health System and Brown University, Providence, RI, United States; ^5^ Cancer Center at Brown University, Thoracic Oncology, Providence, RI, United States; ^6^ Hematology/Oncology Division, Department of Medicine, Lifespan Health System and Brown University, Providence, RI, United States

**Keywords:** SCLC, immunotherapys, chemotherapy, imipridones, genomics

## Abstract

Lung cancer is the second most common cancer in the United States, and small cell lung cancer (SCLC) accounts for about 15% of all lung cancers. In SCLC, more than other malignancies, the standard of care is based on clinical demonstration of efficacy, and less on a mechanistic understanding of why certain treatments work better than others. This is in large part due to the virulence of the disease, and lack of clinically or biologically relevant biomarkers beyond routine histopathology. While first line therapies work in the majority of patients with extensive stage disease, development of resistance is nearly universal. Although neuroendocrine features, Rb and p53 mutations are common, the current lack of actionable biomarkers has made it difficult to develop more effective treatments. Some progress has been made with the application of immune checkpoint inhibitors. There are new agents, such as lurbinectedin, that have completed late-phase clinical testing while other agents are still in the pre-clinical phase. ONC201/TIC10 is an imipridone with strong *in vivo* and *in vitro* antitumor properties and activity against neuroendocrine tumors in phase 1 clinical testing. ONC201 activates the cellular integrated stress response and induces the TRAIL pro-apoptotic pathway. Combination treatment of lurbinectedin with ONC201 are currently being investigated in preclinical studies that may facilitate translation into clinical trials for SCLC patients.

## Introduction

Lung cancer is the second most prevalent cancer diagnosis in the United States and has the highest mortality rate. SCLC comprises approximately 15% of all lung cancers. This is a man-made epidemic caused by cigarette smoking. Cigarette smoke contains polycyclic aromatic hydrocarbons, arsenic, and other potent carcinogens which cause the genetic changes which create SCLC (Govindan et al., 2006). The natural history of SCLC is to grow and spread quickly with a doubling time as short as 25–30 days and unique propensity to hematogenous spread (Pietanza et al., 2015). Patients with SCLC are typically diagnosed with a hilar mass or other bulky lymphadenopathy, frequently accompanied by symptoms of cough and dyspnea. It is uncommon for patients to present with solitary nodules or thoracic lymphadenopathy. Widespread metastatic disease often presents with weight loss, bone pain and neurologic problems. Without treatment, the median survival time for extensive disease is measured in weeks. Limited stage disease has a median survival time of 15–20 months, and the overall 5 years survival rate for SCLC is less than 7% (Byers and Rudin, 2015). The poor survival for patients with SCLC has not changed much in 4 decades (Weisenthal, 1981).

Other than categorizing the disease into limited or extensive stage, SCLC defies practical clinical or genomic categorization, and management decisions are stark. Often, patients are in an urgent or desperate situation upon initial presentation or at the time of recurrence of disease, e.g., superior vena cava syndrome (Chan et al., 1997; Brzezniak et al., 2017). Initial combination chemotherapy is typically, and dramatically effective, but responses are short-lived and recurrent disease is virulent. First-line, platinum-based chemotherapy, typically combined with etoposide, has been the foundation for SCLC treatment over the past half-century, with response rates up to 80% (Pietanza et al., 2015). These therapies also are toxic to patients, with many experiencing hair loss, high-grade fatigue, cytopenias, nausea, and diarrhea.

In response to the dismal prognosis of SCLC, innumerable drugs have been tested, several drugs have earned NCCN compendium listing, and some drugs are FDA-approved. Recent advances in SCLC treatment have been made with immune checkpoint inhibitor therapy (Esposito et al., 2020). Biomarkers such as PD-L1 protein expression, and tumor mutation burden, are not sufficiently robust to select which patients should receive it. Similarly, no biomarkers exist for treatment selection of other available drug treatments. In this complex disease, there exists a gap between preclinical efficacy in treatment and trial outcomes. This is due to the virulence mechanisms of the SCLC cells, as well as resistance to treatment that quickly develops in these patients.

Emerging therapies include novel immune therapies, as well as drugs with innovative mechanisms of action that target specific molecular pathways. Our review will detail some of the promising approaches emerging from a landscape which currently lacks molecular biomarkers for refinement of drug therapy selection.

## Immunotherapy Increases Survival in SCLC

In recent years, immunotherapies have been used to increase overall survival in SCLC (Pavan et al., 2019; Saltos et al., 2020). Increasing tumor-specific T-cell immunity by inhibiting programmed death ligand 1 (PD-L1)–programmed death 1 (PD-1) signaling has shown promise in the treatment of small cell lung cancer, among many other malignancies, and are now routinely added to first-line therapy (Horn et al., 2018; Zhou et al., 2020). PD-1 inhibitors target PD-1 receptors on T-cells and prevent the interaction between PD-1 and its ligands, PD-L1 and PD-L2. PD-1 inhibition enhances T-lymphocyte function and increases cytokine crosstalk between the PD-1 positive T-cells and dendritic cells specialized in activation in the tumor microenvironment. A consequence of this interaction with PD-L1 and PD-L2 is the release of proinflammatory cytokines, such as TNF-α and IFN-γ, which enhance the stem-like properties of T-cells in the tumor microenvironment (Berraondo, 2019). PD-L1 inhibitors, such as atezolizumab, and durvalumab, target the interaction of PD-1 and B7, prevent protumor effects and restore antitumor T-cell function. Additionally, PD-L1 has been shown to exert non-immune proliferative effects on tumor cells (Han et al., 2020) providing an additional benefit to anti-PD-L1 therapies. An important difference between PD-1 inhibitors and PD-L1 inhibitors is that PD-L1 inhibitors still allow the interaction between PD-1 and PD-L2. The continued binding of PD-1 to PD-L2 weakens the immune and proinflammatory response, and, in theory, makes the therapy more tolerable for patients, decreasing the risk of adverse effects.

The CheckMate 032 trial evaluated the efficacy of PD-1 inhibitor nivolumab compared to the combination of nivolumab and ipilimumab, a CTLA-4 inhibitor in patients with previously-treated, extensive stage small cell lung cancer. The combination was shown to have a higher response rate than nivolumab alone (Antonia et al., 2016). In August 2018 the US FDA approved nivolumab for the treatment of patients with metastatic small cell lung cancer whose cancer has progressed after platinum-based chemotherapy and at least one other line of therapy based on the results of CheckMate 032. A subsequent phase 3 study (CheckMate 451), failed to demonstrate the efficacy of ipilimumab and nivolumab when started after initial response to chemotherapy, and off-label use of the combination has fallen out of favor (Owonikoko et al., 2021).

The KEYNOTE-028 study evaluated efficacy of the PD-1 inhibitor pembrolizumab in recurrent SCLC patients. The study showed an overall response rate of 18.7% (Chung et al., 2018). Patients with PD-L1 negative tumors had a median survival of 7.7 months, while PD-L1 positive patients had an impressive overall survival of 14.6 months (Chung et al., 2018), indicating pembrolizumab may have more benefit in patients with PD-L1 positive tumors. It should be noted that FDA approval was withdrawn from nivolumab and pembrolizumab in early 2021.

Atezolizumab, when added to first line carboplatin and etoposide, improves overall survival. The IMpower33 study showed that patients receiving atezolizumab, carboplatin and etoposide had a median overall survival of 12.3 months compared to 10.3 months in the control group (Mathieu et al., 2021). Progression free survival was also statistically improved, with a PFS of 5.2 months in the experimental arm compared to 4.3 months in the control arm (Mathieu et al., 2021). In March 2019, the US FDA approved atezolizumab in combination with carboplatin and etoposide for the first-line treatment of patients with extensive-stage small cell lung cancer.

The CASPIAN study tested another PD-L1 inhibitor, durvalumab, in combination with etoposide and platinum. In this study, there was statistical benefit to adding durvalumab to first-line treatment, with patients receiving durvalumab, etoposide and platinum achieving an overall survival of 13.0 months compared to 10.3 months in the control arm (Mathieu et al., 2021). In March 2020, the US FDA approved durvalumab in combination with etoposide and either carboplatin or cisplatin as first-line treatment of patients with extensive-stage small cell lung cancer. A third study tested platinum-etoposide +/− the anti-PD-1 drug, pembrolizumab, with similar results, although the results in the pembrolizumab study were not statistically significant. (Rudin et al., 2020). There have been no head-to-head comparisons of atezolizumab, durvalumab, or pembrolizumab for this indication, although benefit is similar across studies.

While immunotherapies have had a fair amount of success relative to other treatment options in SCLC in the past 3 years, it is important to note that one of the many virulence factors that make SCLC difficult to target is their innate ability to avoid the surveillance of the host immune system. Compared to NSCLC cells, SCLC cells are less likely to be recognized by the host NK cells and induce an immune mediated response without pharmacological intervention. It has been reported that SCLC cells have a lower expression MHC-1 than their NSCLC counterparts (Zhu et al., 2021). Lack of MHC expression is what can drive reduced immunogenicity, despite SCLC’s high tumor mutational burden (Zhu et al., 2021). Zhu et al. found that when innate immune cell interactions with SCLC were investigated, it was found that MICA/B and ULBP1,2,3 expression was considerably reduced when compared to NSCLC cells (George et al., 2015; Zhu et al., 2021). Similarly, SCLC NKG2DL levels were reduced and the protein expression of NKG2DLs in human SCLC-A lines showed undetectable levels of both MICA/B on their surface and soluble MICA/B (Zhu et al., 2021). These findings indicate that SCLC-A cells may have a diminished visibility to adaptive and innate immune responses. The lack of NKG2DL expression allows SCLC cells to evade the immune response and escape NK surveillance (Ni et al., 2017; Zhu et al., 2021).

This illustrates another important area of potentially beneficial therapy that requires further investigation as it relates to immune surveillance of cancer cells. Pharmacologic strategies of increasing NK-recognition of SCLC cells, including epigenetic regulators, could sensitize the tumor cells to therapeutic agents and to T-cell killing (Zhu et al., 2021). HDAC inhibitors are a promising option to suppress tumor growth and SCLC cell proliferation. These inhibitors also induce Notch signaling and induce cell-cycle arrest, and have been studied extensively *in vitro* (Zhu et al., 2015; Sun et al., 2018). A preclinical HDAC inhibitor showed potential to cause NK-dependent killing of the tumor cells, showing the NK-mediated antitumor effect of shHDAC6, though this model was not related to NKG2DL activity (Liu et al., 2018). Additional studies have shown that NKG2DL stimulating therapies have promoted responses in patients receiving NK-infusions (Cifaldi et al., 2017). This suggests that HDAC inhibitors may provide a synergistic benefit if added to an adoptive NK-cell transfer for patient treatment (Zhu et al., 2021). Lack of NKG2DLs can potentially be exploited by future studies to evaluate NK-cell activation therapies in SCLC treatment investigations (Zhu et al., 2021), especially those that monitor expression of NK activating ligands such as NKG2DLs.

These studies confirm that there is significant benefit to adding PD1/PD-L1 inhibitors to first-line chemotherapy in extensive stage SCLC, while also underscoring the importance of investigating epigenetic regulators in SCLC. It is important to note, that research to enhance the ability of the immune system to kill SCLC and its inherent properties are a major research effort. While advances in immunotherapy in SCLC patients are modest and there has not been an overwhelming breakthrough with these therapies, the effects of immunotherapies remain statistically significant and clinically relevant.

## Genomics of Small Cell Lung Cancer

SCLC arises from stem cells that partially differentiate to have a neuroendocrine phenotype resulting in a unique appearance under the microscope. The histopathology of SCLC often shows dense sheets of small cells with neuroendocrine features (Bernhardt et al., 2016) that divide quickly and show frequent mitosis and high nuclear to cytoplasm ratio with little to no nucleoli (Bernhardt et al., 2016). These features contribute to SCLC cells’ ability to proliferate, migrate, and form both local and metastatic tumors. The genetic profile of SCLC predisposes the cells to have an aggressive nature, and the multitude of mutations and high mutational burden in SCLC makes treating with chemotherapy a difficult task. It is essential to note that certain SCLC subsets will contain heterogeneity and contain non-small cell lung cancer features (Gazdar et al., 2017). Additionally, 15% of all SCLC do not express neuroendocrine markers at all (Gazdar et al., 2017) and may have protein expression patterns more similar to adenocarcinoma of the lung.

The arrival of combination chemotherapy has significantly improved pharmacologic effectiveness when treating SCLC. However, due to the complex genetic profile of SCLC, targeted chemotherapies remain elusive. Targeted therapies pursue specific genes and proteins that are involved with the growth and survival of tumor cells. These therapies can block the tumorigenic effects of specific abnormalities that make SCLC cells grow, survive, and metastasize. These therapies consist of small-molecule drugs that block angiogenesis and cell proliferation or monoclonal antibodies that block a specific target on the exterior of cancer cells. Other targeted therapies include apoptosis-inducing drugs, angiogenesis inhibitors and immunotherapies. Despite the diversity of actions of the drugs and the virulence mechanisms they target, targeted therapies in SCLC have yielded disappointing results.

Many prominent genomic features of small cell lung cancer currently lack drug therapy alternatives. For example, approximately 27% of SCLC has a SOX2 amplification (Gadgeel, 2018) SOX2, which encodes a transcriptional regulator of stem cells, promotes initiation and growth of SCLC (Gazdar et al., 2017) SOX2, located on chromosome 3q26.3-q27 is implicated in SCLC by a multiplication of the 3pq26.3 gene locus (Kaufhold et al., 2016). However, there are currently no therapies for combatting the effects of SOX2 amplification and its tumorigenic effect on initiating SCLC occurrence and growth.

Prominent phenotypic features of small cell lung cancer also lack drug therapy alternatives. For example, SCLC cells growing *in vitro* demonstrate that adhesion to extracellular matrix (ECM) increases tumorgenicity and resistance to chemotherapeutic agents. This is a result of B1 integrin-stimulated tyrosine kinase activating and inhibiting chemotherapy induced apoptosis (Sethi et al., 1999). The ECM protects SCLC cells from chemotherapy induced apoptosis. When the cells adhere to laminin (Ln), fibronectin (Fn) or collagen IV, there is substantial protection from chemotherapy-induced apoptosis, as was shown in H69, H345 and H510 cell lines (Sethi et al., 1999). Adhesion of the H69 cell line to Ln stimulates PTK activity and prevents chemotherapy induced caspase activation in the apoptosis pathway (Sethi et al., 1999). To target this phenotype, there has been great hope that matrix metalloproteinase inhibitors might hinder the virulent effects of the SCLC-ECM interaction. However, in two randomized trials: one with marimastat, the other with tanomastat, the results were disappointing, as neither survival nor quality of life improved (Shepherd et al., 2002; Zhang and He, 2013).

### Tumor Suppressor Gene Loss

**FIGURE 1:**
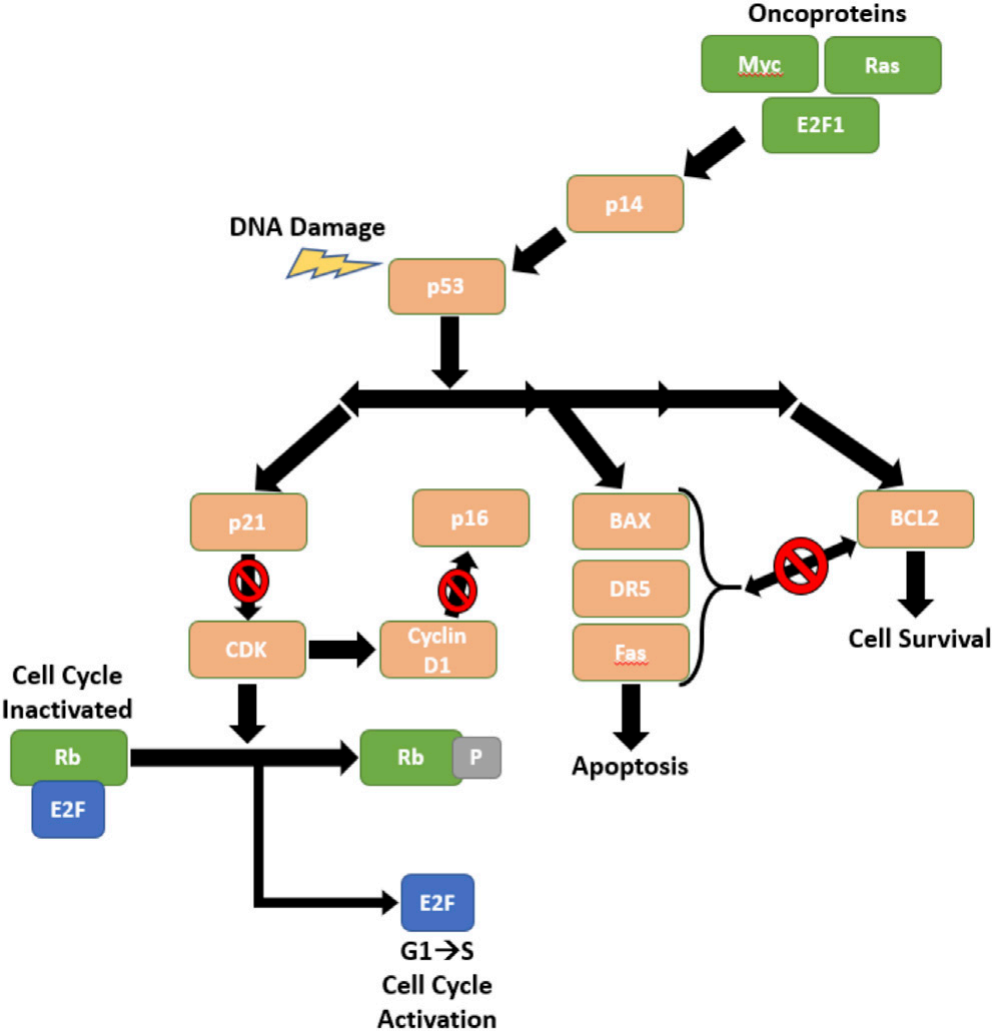
The Role of p53 and Rb in Regulating Cell Cycle Progression.

The loss of function of tumor protein 53 (P53) (Byers and Rudin, 2015) is almost universal in SCLC patients, as P53 is mutated in over 90% SCLC diagnoses (Wistuba et al., 2001). Additionally, Retinoblastoma 1 (RB1) (Helin et al., 1997) is frequently deleted in SCLC. Both P53 and RB1 are tumor suppressor genes that encode proteins that regulate the cell cycle and cell survival. Figure 1 displays the mechanisms of p53 in response to DNA damage in SCLC cells. These are occasionally accompanied by a 3p deletion (Whang-Peng et al., 1982) and when they are deleted, the mechanism indicated in Figure 1 is not able to inhibit cell growth or uncontrolled cell proliferation, and consequently there is tumor growth and disease progression. P53 and RB1 deficient SCLC tumors may also express increased cKit (Rao et al., 2020), MYC amplification (20% of patients (Pietanza et al., 2015)), and loss of phosphatase and tensin homolog (PTEN).

In the RB1 and P53 deficient SCLC cells, the Hedgehog pathway, a cell-intrinsic pathway, further promotes tumorigenicity. Activation of the Hedgehog signaling molecule promotes clonogenicity of SCLC *in vitro* and accelerates the initiation and progression of SCLC in mice *in vivo*. (Park et al., 2011). Further, suppression of Hedgehog signaling molecules inhibited growth of both mouse and human SCLC cells (Park et al., 2011). Hedgehog pathway inhibitors target certain Hedgehog activators and have been tested as an addition to combination chemotherapy. These inhibitors work by a mechanism of action that inhibits Smoothened, a Hedgehog pathway activator (Gadgeel, 2018) Vismodegib, a Smoothened inhibitor, was added to chemotherapy regimens in late-stage SCLC, but the combination did not show any overall benefit (Belani et al., 2016). Also of note, that, in the same study, cixutumumab, an IGF-1R inhibitor, was tested and likewise did not show any benefit when added to standard chemotherapy regimens for all tumor types.

In RB1 deficient tumor cells, apoptosis evasion mechanisms also add to the complexity of the SCLC genome. RB1 deficient cells can have overexpression of HIF-1α and aid cancer cells in evading apoptosis. HIF-1α over expression also aids in cell migration, increasing metastasis and promotion of angiogenesis *via* upregulation of VEGF (Forsythe et al., 1996). There has been significant progress in treating Rb-deficient tumors, as therapies targeting HIF-1α have shown preclinical antitumor efficacy (Zhao et al., 2020). In Rb deficient cells, such as many SCLC genotypes, there has been a significant benefit, *in vivo*, to dual inhibit CDK4/6 and HSP90 (Zhao et al., 2020). CDK4/6 and HSP90 inhibition suppress the tumorigenic traits of HIF-1α. This has been shown to significantly inhibit tumor cell viability in RB1 deficient colorectal cancer cell lines (Zhao et al., 2020), though the model is applicable in any RB1 deficient cancer cell.

Apoptosis/programmed cell death regulation also plays a significant role in SCLC virulence. Variations in the apoptosis cascade can increase SCLC progression and lead to worse prognosis for patients. SCLC has been further found to avoid chemotherapy-induced apoptosis by upregulation of BCL2, an anti-apoptotic gene (Gazdar et al., 2017). While BCL2 is an anti-apoptotic gene, BAX is its proapoptotic counterpart (Brambilla and Gazdar, 2009). SCLC cells are able to escape apoptosis *via* variations in the BCL2:BAX balance.

BCL2 inhibitors have been studied as an option to combat SCLC resistance. BCL2 inhibitors target the antiapoptotic characteristics of SCLC cells, as an elevated level of BCL2 is an indicator of poor prognosis. (Lawson et al., 2010). However, despite early indications of success, Obatoclax, a BCL2 inhibitor, did not improve any clinical endpoints when combined with carboplatin and etoposide in a randomized study. (Langer et al., 2014). The oral BCL2 inhibitor, AT-101 also did not show any improvement in overall survival and the study was terminated at the first interim analysis. (Baggstrom et al., 2011). It is thought that these initial agents may lack potency in against BCL2, preventing a synergistic response when added to chemotherapy combinations and that they may be further studied at more potent dosages.

### Oncogenic Drivers

All three members of the MYC family of genes, MYC, MYCL, MYCN are amplified in SCLC cell lines. Pulmonary neuroendocrine cells with deleted copies of RB1, P53, and P130 grow and express neuroendocrine marker genes but do not proliferate and become tumors. (Gazdar et al., 2017). Conversely, the addition of MYC family members, particularly MYCL, to the neuroendocrine cells with the previously mentioned deletions rapidly results in tumorgenicity (Gazdar et al., 2017).

Aberrant amplification of MYC family genes in SCLC can be explained by mechanisms including, promoter activation, attenuation of transcription, and control of gene copy number. In cells, each MYC family gene uses a different combination of these mechanisms to control mRNA transcription. In the context of SCLC, MYC has been shown to be regulated by both initiation and attenuation of transcription, MYCN appears to be regulated at the level of initiation of transcription, and MYCL has been shown to be regulated by attenuation of transcription (Krystal et al., 1988).

In normal cells, MYC expression is tightly regulated by transcription factors CNBP, FuBP1, and TCF as well as structural DNA elements (Brägelmann et al., 2017). It has been observed that phosphorylation of both MYC and MYCN affects polyubiquitination and by extension, proteasomal degradation, but no such regulatory mechanism has been reported for MYCL (Sjostrom et al., 2005; Malempati et al., 2006; Brockmann et al., 2013).

In mouse models, MYC withdrawal has led to tumor regression, indicating that MYC may be a valuable target in SCLC. This, however, has proven difficult, as MYC drivers are not due to intrinsic oncogenic mutations that could easily be targeted by therapeutic agents (Fletcher and Prochownik, 2015; Brägelmann et al., 2017). Rather, they are activated due to overamplification. This makes compound discovery aiming at kinase inhibition, such as agents that target mutated proteins only, extremely difficult (Brägelmann et al., 2017).

### DNA Repair

Overexpression of DNA repair proteins such as PARP1, checkpoint kinase 1 (Chk1), and enhancer of zeste two polycomb repressive complex two subunit (EZH2) are independent of DNA-level mutations, but significantly contribute to tumor growth (Byers and Rudin, 2015). SCLC tumors also induce FGFR family alterations, and some have demonstrated sensitivity to FGFR inhibitors. However, the relationship between SCLC mutations and the family pathways is not currently known (Byers and Rudin, 2015).

SCLC relies on the ATR-CHK1 pathway to overcome cellular stress during the replication process that would otherwise cause DNA damage (Hsu et al., 2019). Due to the higher concentration of Chk1 gene expression and protein in SCLC compared to NSCLC, Chk1 inhibitors are a useful therapeutic agent in attempting to hinder this potent virulence mechanism in SCLC. Chk1 inhibitors such as prexasertib demonstrated effectiveness in SCLC cells *in vivo*, genetically engineered mice (GEM) and chemo-resistant mouse models (Sen et al., 2017). Chk1 showed synergistic effects when administered with cisplatin to induce mitotic cell death, combating resistance that may develop to first line therapies (Hsu et al., 2019).

PARP inhibitors such as Olaparib, Veliparib, Talazoparib et al. target PARP1 overexpression *via* the biomarker SLFN11 and work by two mechanisms. First, trapping PARP to the single strand DNA breaks and preventing repair. Second, PARP inhibitors inhibit poly ADP-ribosylation (PARylation) and binding of PARP to DNA (Murai et al., 2012), thus, preventing DNA repair and allowing for apoptosis pathways to begin their cascade and induce cell death. A phase I trial with Talazoparib showed some activity in decreasing chemotherapy resistance, including SCLC tumors (de Bono et al., 2017). These patients achieved an overall response rate of 9.0% with the duration of the response lasting up to 15 weeks. (de Bono et al., 2017).

The proteins of the Schlafen family are involved in regulation of cell proliferation and possibly helicase activity. EZH2 amplification increases resistance to chemotherapy by downregulation of Schlafen 11 (SLFN11) expression (Gazdar et al., 2017). EZH2 downregulates SLFN11 by histone modification and methylation in SCLC. EZH2 inhibition has become an important target for therapeutics as it was shown to decrease resistance in cells treated with cisplatin (Berns and Berns, 2017).

A 2015 study examined the efficacy of temozolomide and the PARP inhibitor, veliparib, in SCLC. In their phase II, double-blind trial, 4 month progression-free survival and overall survival did not differ between the treatment and placebo arms, though a significant overall response was found in the temozolomide/veliparib arm (Pietanza et al., 2018). Additionally, it was found that SLFN11 expression was associated with improved progression-free and overall survival in the temozolomide/veliparib arm (Pietanza et al., 2018).

DNA damage repair by cancer cells is another mechanism through which resistance occurs in SCLC cells. DNA repair can occur due to overexpression of repair proteins such as PARP1, wee-like protein kinase 1 (WEE1), Chk1, Rad3-related protein (ATR) and ataxia telangiectasia mutated (ATM) protein kinase (Pietanza et al., 2015; Berraondo, 2019).

SCLC resistance to the platinum series of drugs occurs, in part, due to the repair of DNA damage (Goldstein and Kastan, 2015) *via* glutathione (GSH). GSH is prevalent in SCLC tumors and enhances the repair of DNA damage caused by drugs such as cisplatin, as well as increasing inactivation of the drug before it reaches the DNA (Chen et al., 2020). Various research studies have reinforced this relationship. In 1990, Meijer, Mulder et al. confirmed the relationship between GSH and cisplatin resistance in SCLC cells (Meijer et al., 1990).

Resistance in SCLC cells has also been indicated by abnormal DNA methylation. In 2017, Gardner et al. examined the mechanism of resistance by SCLC tumors to cisplatin and etoposide, a common chemotherapy combination, and revealed that histone H3 lysine 27 trimethylation (H3K27me3) resulted in resistance to the cisplatin and etoposide. (Gardner et al., 2017).

### Cell Cycle and Differentiation Mechanisms

Notch signaling, while a powerful protumor effector in most cells, is uniquely diminished in the majority of SCLC cells. In SCLC, Notch acts as a tumor suppressor by negatively regulating neuroendocrine differentiation (Gazdar et al., 2017). SCLC cells express Notch inhibitors delta like non-canonical Notch ligand 1 (DLK1) and DLL3 (DLL3 is expressed as a neoantigen on the surface of some SCLC cells) or have inactivating mutations in the Notch pathways (Gazdar et al., 2017), leading to inactivation of the tumor suppression.

Inactivation of the Notch pathway in SCLC promotes tumor growth and metastases, and results in worse prognoses. In approximately 80% of SCLCs, Notch ligand Delta-like protein 3 (DLL3) is upregulated to act as an inhibitor of the pathway (Chapman et al., 2011). The TRINITY trial in 2018 evaluated the effectiveness of rovalpituzumab tesirine (ROVA-T), an antibody drug conjugate, in patients with recurrent SCLC with high DLL3 expression. There was a marginal benefit to patients receiving this drug (Carbone et al., 2018), though severe toxicities occurred in 40% of patients (Gadgeel, 2018), suggesting the modest benefit is outweighed by adverse reactions in a considerable portion of the studied patient population.

A significant portion of SCLC cells express the transcription factor achaete-scute homologue 1 (ASCL1), which enhances the survival and growth of these cells and ASCL1 amplified cells express the full set of neuroendocrine markers (Gazdar et al., 2017). Often associated with ASCL1, approximately 15% of SCLC cells express neurogenic differentiation factor 1 (NEUROD1) (Gazdar et al., 2017), a master regulator that enhances cell proliferation and growth.

This subgroup of SCLC, with mutated ASCL1, is characterized by having faster growth rates, MYC amplification, and oncogenic transcription regulation. Alisertib, an aurora kinase A/B inhibitor, has shown tumor suppression in SCLC cells with variant ASCL1 and MYC amplification in preclinical studies (Mollaoglu et al., 2017). Alisertib was added in clinical studies to regimens consisting of weekly paclitaxel in patients with recurrent SCLC. There was some improvement in overall survival for these patients but not enough to reach statistical significance (Gadgeel, 2018). However, in 46 patients whose tumors were analyzed *via* immunohistochemistry, patients with positive MYC expression had significant benefit in progression free survival, while the patients with MYC negative expression actually had worse outcomes when alisertib was added to paclitaxel (Gadgeel, 2018). It is important to note that these results have not been repeated or confirmed in prospective trials.

## ONC201: A Promising Novel Agent for SCLC

There are currently over 275 clinical trials registered with the FDA in SCLC that are active or recruiting patients for study. This large number is due, in part, to a rapid, trial-and-error approach which has evolved to apply any and all novel therapies to patients diagnosed with a highly prevalent and desperate disease. While there has been a disappointment in the availability of targeted therapies available to treat SCLC patients, there is optimism in the form of new therapies being tested.

ONC201 is an emerging imipridone therapy that has demonstrated strong antitumor properties *in vivo* and *in vitro* (Prabhu et al., 2020). It is entering an increasing number of clinical trials as it has effective tumor killing ability, while being well tolerated. ONC201 is administered weekly at a recommended phase two dose of 625 mg based on pharmacokinetics and pharmacodynamics in phase 1 testing, and preclinical dose intensification studies (Wagner et al., 2018). ONC201 activates the integrated stress response, (Kline et al., 2016), resulting in reduced proliferation of SCLC tumor cells (Lee et al., 2020). Currently being studied is the possibility of adding ONC201 to combination chemotherapy regimens, targeting the DNA of the tumor cells along in addition to activating the integrated stress response, thus, inducing higher levels of cleaved PARP, resulting in a synergistic pro-apoptotic effect.

Tests *in vivo* in SCLC and in clinical trials in additional solid tumors, show that ONC201 works by increasing cellular stress and inducing TRAIL-mediated apoptosis in the p53 pathway in several tumor cell lines. TRAIL is an endogenous protein that induces tumor cell apoptosis *via* its interaction with death receptors DR4 or DR5 (Ashkenazi, 2002). TRAIL is expressed in many human tissues, including lung tissue, as well as certain immune cells following cytokine activation (Fanger et al., 1999; Griffith et al., 1999; Allen et al., 2015).

ONC201 works by a unique mechanism of action, inhibition of dopamine receptors and direct activation of the enzyme ClpP (Prabhu et al., 2020). ClpP is allosterically modified by ONC201 to open substrate channel areas and alter the conformation of its active site (Prabhu et al., 2020). This allosteric modification causes hyperactivation of ClpP’s proteolytic activity, leading to degradation of subunits in the electron transport chain, involved in cell respiration. This degradation results in impairment of oxidative phosphorylation and causes elevated cell stress levels, triggering apoptosis. While the mechanisms of the interaction between ClpP and the mitochondrial mechanics remain to be investigated, ClpP inactivation has made tumor cells at least partially resistant to ONC201 in AML, acute lymphoblastic leukemia (ALL) and breast cancer cells (Prabhu et al., 2020).

Two pathways are consistently impacted by ONC201 administration—activation of the ISR pathway (Kline et al., 2016; Prabhu et al., 2020) and Akt/ERK inactivation (Allen et al., 2013; Prabhu et al., 2020). The ISR pathway is also activated by proteasome inhibitors, but when activated by ONC201, causes upregulation of ATF-4 translation and CHOP transcription rapidly in tumor cells (Kline et al., 2016). While ISR activation happens rapidly, Akt/ERK inactivation happens over 2–3 days (Allen et al., 2013). These effects combine to produce a powerful upregulation of TRAIL, a proapoptotic ligand, *via* activation and nuclear translocation of Foxo3a and its receptor DR5 (Prabhu et al., 2020). It is noteworthy that DR5 is induced by activation of ATF4 and CHOP through the integrated stress response (Kline et al., 2016). An additional mechanism ONC201 may be effective in killing tumor cells is the degradation of MYC through a proteasomal pathway involving GSK3β mediated phosphorylation of threonine 58 (Ishida et al., 2018).

One of the most attractive aspects in the prospect of ONC201 as an emerging SCLC therapy is its low risk of toxicity when administered in both mice and humans (Prabhu et al., 2020). ONC201 displays a synchronization of unique anti-tumor mechanisms *via* early-stage ISR activation in the tumor cells that seemingly spares healthy epithelial and organ tissue cells (Allen et al., 2016). This remains the case in SCLC cells, ONC201 does not exhibit cytotoxic effects. While ONC201 was shown to effectively inhibit tumor cell survival in a dose-dependent experiment in the cell line H460 (Feng et al., 2016). ONC201 was also tested in patient derived human-cells and was found to inhibit tumor cell survival, while remaining nontoxic to healthy lung epithelial cells and healthy hepatocytes, indicating ONC201s specificity for tumor cells in lung tissue (Feng et al., 2016). ONC201 was also tested *in vitro* in mouse models and did not produce any significant toxicities in the mammals and did not induce DR5 or TRAIL in normal epithelial cells (Feng et al., 2016).This could potentially be related to the fact that Akt and Erk expression is low in healthy cells, therefore without any Akt and Erk inhibition, DR5 and TRAIL are not induced (Feng et al., 2016).

While approximately 10% of all SCLC patients present with brain metastases and 40% of all SCLC patients will experience brain metastases in their disease, it is crucial to consider which novel agents may cross into the central nervous system. (Schuette, 2004). The blood-brain barrier becomes less relevant in these patients, as it has most likely already been compromised due to the invasive nature of the disease (Schuette, 2004). ONC201 shows activity against tumors within the CNS. ONC201 has already shown its preclinical efficacy in brain tumors such as gliomas, and it is currently involved in clinical trials treating gliomas and various metastatic neuroendocrine tumors, where it has shown impressive clinical efficacy. These trials are displayed in Table 1 **.** The small molecule therapy crosses the blood-brain barrier and has showed efficacy against glioblastomas, including those resistant to the standard of care temozolomide (Ralff et al., 2017). In addition to the clinical evidence with ONC201 that has accumulated since 2014, (Prabhu et al., 2020), mouse models have clearly displayed the efficacy of ONC201 crossing the blood-brain barrier and inhibiting tumor growth, due to dual inactivation of Akt and ERK and activation of the integrated stress response (Allen et al., 2013; Kline et al., 2016; Wagner et al., 2018). This further validates the investigation of ONC201 for SCLC cells, both primary and metastatic tumors, regardless of their location in the body.

**TABLE 1:** Current FDA Trials involving ONC201 (clinicaltrials.gov).

Name of study	Phase	Disease	Status	ClinicalTrials.gov identifier
ONC201 in Relapsed/Refractory Acute Leukemias and High-Risk Myelodysplastic Syndromes (HR-MDS)	I/II	Leukemia	Active, not recruiting	NCT02392572
Oral ONC201 in Recurrent GBM, H3 K27M Glioma, and Midline Glioma	II	Various Gliomas	Recruiting	NCT02525692
Oral ONC201 in Relapsed/Refractory Multiple Myeloma	I/II	Multiple Myeloma	Active, not recruiting	NCT02863991
Phase 2 Study of ONC201 in Neuroendocrine Tumors	II	Recurrent/Metastatic Neuroendocrine Tumor	Recruiting	NCT03034200
ONC201 in Adults With Recurrent H3 K27M-mutant Glioma	II	Glioma	Recruiting	NCT03295396
ONC201 in Recurrent/Refractory Metastatic Breast Cancer and Advanced Endometrial Carcinoma	II	Triple Negative Breast Cancer, Endometrial Cancer, Hormone Receptor Positive, HER2 Negative Breast Cancer	Active, not recruiting	NCT03394027
ONC201 in Pediatric H3 K27M Gliomas	I	Glioma, Diffuse Intrinsic Pontine Glioma	Recruiting	NCT03416530
ONC201 in Recurrent or Metastatic Type II Endometrial Cancer Endometrial Cancer	II	Recurrent Endometrial Cancer	Recruiting	NCT03485729
BrUOG 379 phase Ib/II Trial ONC201 + nivolumab in MSS mCRC (379)	I/II	Metastatic Colorectal Cancer	Active, not recruiting	NCT03791398
ONC 201 Maintenance Therapy in Acute Myeloid Leukemia and Myelodysplastic Syndrome After Stem Cell Transplant	I	AML	Recruiting	NCT03932643

ONC201 is particularly effective in SCLC due to the induction of TRAIL and DR5, as well as activated caspase-8, which induces extrinsic apoptosis (Prabhu et al., 2020). The drug also works as a selective competitive and competitive D2 receptor (DRD2) antagonist (Prabhu et al., 2020), which is overexpressed in SCLC patients who often have elevated plasma dopamine levels. Further, dopamine is a critical regulator in the innate and adaptive immune systems. Dopamine receptors are expressed by both T-cells and NK cells that can modulate the immune response to tumor formation and growth (Zhao et al., 2013; Wang et al., 2019), as dopamine is a negative regulator of NK response (Mikulak et al., 2014). DRD2 receptor inhibition has been shown to activate NK cells in the immune response to tumor cells (Mikulak et al., 2014), however, these mechanisms have not been evaluated in mouse or human models. ONC201 has been shown to activate NK cells (Wagner et al., 2018) and has clinical activity in neuroendocrine tumors such as pheochromocytoma and paraganglioma (Anderson and Gortz, 2021). This preclinical data is the basis for continued investigation, as ONC201 is under further review in a range of neuroendocrine and metastatic tumors (Anderson and Zahler, 2020). There is also evidence for synergy between ONC201 and EZH2 inhibitors in a variety of tumor types and this may be relevant to SCLC as discussed earlier (Zhang et al., 2021). Table 1 displays current FDA approved clinical trials involving ONC201 (clinicaltrials.gov).

## 5 Lurbinectidin: A Novel Agent in Clinical Use in SCLC

In June 2020, the FDA granted accelerated approval to lurbinectedin for patients with extensive stage SCLC with disease progression while receiving or after treatment with platinum-based chemotherapy. A phase two study treated 105 previously-treated patients, and demonstrated an overall response rate of 35% (95% CI 26–45%), which includes an impressive 22% response rate in 45 patients whose cancer started to grow <90 days since last dose of platinum (platinum-refractory disease), with a median duration of response of 5 months. Treatment was well tolerated, with the most common high-grade side effects being anemia (9%), neutropenia (46%), and thrombocytopenia (7%), and there were no treatment-related deaths (Trigo et al., 2020).

Lurbinectedin is an inhibitor of RNA polymerase II, an enzyme commonly hyperactivated in SCLC, and induces DNA breaks in cells that result in apoptosis (Markham, 2020). The drug covalently binds to central guanine in trinucleotide triplets in the minor groove of DNA, forming adducts capable of inducing DNA double-strand breaks (Markham, 2020). It may also induce immunogenic cell death and increase anti-tumor immunity. Lurbinectedin also has an impact on the tumor microenvironment as it is associated with a reduction in tumor associated macrophages (Markham, 2020). Lurbinectedin has been shown to downregulate ASCL1 and thus decrease the rapid growth rate that is characteristic of SCLC cells (Markham, 2020).

The recently completed phase III ATLANTIS trial, evaluating lurbinectedin + doxorubicin compared with cyclophosphamide + doxorubicin + vincristine (CAV) or topotecan enrolled 613 previously-treated patients and will add significant clinical evidence of lurbinectedin’s effects. The ATLANTIS trial was terminated as it missed primary endpoints with patients. Though the combination of doxorubicin and lurbinectedin did not provide positive results, the study did confirm the tolerability and overall activity in patients.

Lurbinectedin is also under investigation for other solid tumors. Table 2 shows currently active or recruiting clinical trials in SCLC, involving lurbinectedin (clinicaltrials.gov).

**TABLE 2:** Current FDA Trials involving Lurbinectedin in SCLC (clinicaltrials.gov).

Name of study	Phase	Status	ClinicalTrials.gov identifier
Study to Assess Safety, Tolerability, Efficacy of PM01183 and atezolizumab in Patients w/Advanced Small Cell Lung Cancer	I	Recruiting	NCT04253145
Lurbinectedin (PM01183) Combined With pembrolizumab in Small Cell Lung Cancer. (LUPER)	I/II	Recruiting	NCT04358237
Immune Checkpoint Inhibition With Lurbinectedin Relapsed/Recurrent SCLC	I/II	Recruiting	NCT04610658

## Discussion

SCLC is a deadly and devastating disease for patients. Many patients present in the advanced stages of disease with distant metastasis. Another complication of SCLC, which makes it even harder to effectively treat, is the absence of targetable biomarkers. Due to the complex genomic profile of SCLC and its numerous virulence mechanisms, resistance to first line chemotherapies inevitably develops, leaving patients with dismal outlooks as the disease progresses and further metastasizes. Targeted therapies in SCLC have been largely unsuccessful.

In the past 3 years, the overall survival of patients with SCLC has been improved with routine, first-line use of anti-PD1/PD-L1 immunotherapy. With over 275 clinical trials either active or recruiting that focus on SCLC, there is optimism in emerging therapeutics. Interestingly, nonplatinum-based therapies that appear to have sufficient efficacy but are much less toxic to patients. ONC201 and lurbinectedin, while mechanistically dissimilar, display effective anti-tumor characteristics while remaining tolerable when administered clinically. These therapies will be important to monitor going forward, as they are further evaluated in clinical trials, as well as *in vivo* and *in vitro*.

The p53 pathway is an important regulatory pathway that is often broken in SCLC. DNA damage or stimuli from oncoproteins can trigger p53 to induce cell cycle arrest, preventing cells from replicating with mutations or damaged DNA. This pathway also exerts influence over the TRAIL pathway *via* its control over DR5, which induces apoptosis (Brambilla and Gazdar, 2009). Figure adapted by way of Brambilla and Gazdar (2009).

## Data Availability

The original contributions presented in the study are included in the article/Supplementary Material, further inquiries can be directed to the corresponding authors.
